# High-Resolution NMR Studies of Human Tissue Factor

**DOI:** 10.1371/journal.pone.0163206

**Published:** 2016-09-22

**Authors:** Kristin M. Nuzzio, Eric D. Watt, John M. Boettcher, Joshua M. Gajsiewicz, James H. Morrissey, Chad M. Rienstra

**Affiliations:** 1 Department of Chemistry, University of Illinois at Urbana-Champaign, Urbana, Illinois, United States of America; 2 Department of Biochemistry, University of Illinois at Urbana-Champaign, Urbana, Illinois, United States of America; 3 Center for Biophysics and Computational Biology, University of Illinois at Urbana-Champaign, Urbana, Illinois, United States of America; University of Pittsburgh School of Medicine, UNITED STATES

## Abstract

In normal hemostasis, the blood clotting cascade is initiated when factor VIIa (fVIIa, other clotting factors are named similarly) binds to the integral membrane protein, human tissue factor (TF). The TF/fVIIa complex in turn activates fX and fIX, eventually concluding with clot formation. Several X-ray crystal structures of the soluble extracellular domain of TF (sTF) exist; however, these structures are missing electron density in functionally relevant regions of the protein. In this context, NMR can provide complementary structural information as well as dynamic insights into enzyme activity. The resolution and sensitivity for NMR studies are greatly enhanced by the ability to prepare multiple milligrams of protein with various isotopic labeling patterns. Here, we demonstrate high-yield production of several isotopically labeled forms of recombinant sTF, allowing for high-resolution NMR studies both in the solid and solution state. We also report solution NMR spectra at sub-mM concentrations of sTF, ensuring the presence of dispersed monomer, as well as the first solid-state NMR spectra of sTF. Our improved sample preparation and precipitation conditions have enabled the acquisition of multidimensional NMR data sets for TF chemical shift assignment and provide a benchmark for TF structure elucidation.

## Introduction

Proteins involved in the blood coagulation cascade are of vital biomedical importance, and understanding the mechanism of blood clotting protein interactions is crucial to the continued development of successful treatments for blood coagulation disorders such as hemophilia, von Willebrand disease, and thrombosis. The blood clotting cascade is initiated through the extrinsic pathway when the activated serine protease, factor VII (fVIIa), binds to human tissue factor (TF) (Factors are named with Roman numerals and the suffix “a” to indicate the activated state.) [1]. A series of other clotting factors are subsequently activated; for example, the membrane-bound TF/fVIIa complex in turn activates fX and fIX via limited proteolysis, and the cascade eventually concludes with clot formation.

Human TF is a type-I integral membrane protein that is expressed on the surface of cells outside the vasculature [2]. It is a member of the class II cytokine receptor superfamily and consists of three domains: the extracellular domain (residues 1–219), a membrane spanning helix (residues 220–242), and the cytoplasmic domain (residues 243–263). The extracellular domain comprises two fibronectin type-III domains, which have primarily β-sheet secondary structure. The isolated extracellular domain of TF (also known as soluble TF, sTF) does not occur naturally, but because it is highly water soluble while retaining the ability to form an active enzyme complex with fVIIa [3], it has been used in a variety of biophysical studies to investigate TF [4–9]. Apart from blood coagulation, TF has also been implicated in a variety of processes, including inflammation, metastasis, and cell signaling [10].

The structure of sTF has been characterized using X-ray crystallography and solution NMR spectroscopy [6–9,11]. Several X-ray crystal structures of sTF exist at high resolution (1.69–2.40 Å) [6–8]; however, some of these structures lack electron density in regions that are hypothesized to interact with fVIIa, fIX, fX, and the membrane bilayer. Solution NMR spectroscopy has been utilized to investigate sTF, and Boettcher et al report site-specific chemical shift assignment for 90% of the protein backbone, including 97% of the amide resonances [11].

Solid-state NMR enables the study of membrane proteins in their native lipid environment. Additionally, there is no fundamental limit on the molecular weight of the protein, although larger proteins present data interpretation challenges. In solution NMR, the slow tumbling times of the protein prohibit larger molecules from being studied; however, in the solid state, linewidths and peak intensities do not depend on the molecular weight of the protein. Solid-state NMR also does not require single crystals as would be needed in X-ray crystallography. Recently developed 3D and 4D magic-angle spinning (MAS) solid-state NMR spectroscopic techniques can provide site-resolution throughout entire uniformly ^13^C,^15^N-labeled proteins, as well as facilitate the chemical shift assignment process [12–16]. Additionally, selective glycerol labeling schemes using either 1,3-^13^C-glycerol or 2-^13^C-glycerol have been employed to further remove chemical shift degeneracy in solid-state NMR spectra [17–19].

A protocol for the expression and purification of ^15^N-labeled sTF employing *S*. *cerevisiae* or inclusion bodies from *E*. *coli* has been reported [9]. We now report a highly efficient protocol for expression and purification of doubly- and triply- (^2^H, ^13^C, ^15^N) labeled and glycerol (1,3-^13^C or 2-^13^C) labeled sTF in the periplasmic space of *E*. *coli*, which allows for the formation of disulfide bonds, thereby obviating the need for protein refolding as previously described [9]. As both solution and solid-state NMR require milligram quantities of pure protein for spectral acquisition, a robust and effective expression and purification protocol for this system is crucial for sTF structural characterization using NMR. Here, we utilize solid-state NMR to evaluate sTF samples as a precursor to examining the TF structure as a whole and demonstrate the benefits of uniform and glycerol labeling schemes on spectral resolution and sensitivity.

## Materials and Methods

### Expression of doubly-labeled (^13^C,^15^N) recombinant sTF

Media used for the expression of each variety of isotopically labeled sTF described in this study are listed in Table 1. All flasks and media were autoclaved or filter-sterilized where applicable prior to use. A C-terminal His-tagged construct of sTF was cloned into plasmid pJH677 with kanamycin resistance and expressed using T7 Express *E*. *coli* cells (New England BioLabs, Inc., Ipswitch, MA, USA). A glycerol stock of transformed cells was used for all subsequent expressions. The sTF glycerol stock was grown on a Luria Broth (LB) agar plate containing 50 μg/mL kanamycin and incubated for ~15 hours at 37°C. A single colony was inoculated into 25 mL modified Studier [20] media (MDG) in a 250 mL baffled flask and grown for 7 hours at 37°C at 300 rpm. The preculture (1 mL) was added to 250 mL MDG in a 2-L baffled flask and grown for ~12–15 hours at 37°C at 300 rpm until the A_600_ of the culture was between 5–7. The cells were harvested by centrifugation for 20 minutes at 25°C at 7000 x g and were subsequently resuspended in 1-L MBG modified Studier media with BioExpress (Cambridge Isotope Laboratories, Inc., Andover, MA, USA) as given in Table 1, to achieve a resulting A_600_ of ~2. The culture was divided into four 2-L flasks and grown for ~30 minutes at 25°C at 300 rpm until an A_600_ of ~3 was reached to deplete any remaining natural abundance isotopes. Expression of sTF was induced with isopropyl β-D-1-thiogalactopyranoside (IPTG) at a final concentration of 100 μM for 24 hours at 25°C. Cells were harvested via centrifugation for 35 minutes at 25°C at 8500 x g. The cell pellets were stored at -80°C prior to purification.

**Table 1:** Components of media used for isotopically labeled growths of sTF.

Natural Abundance (n.a.) Media		Uniform (U) & Glycerol Labeled Media			Deuterated Media		
*n*.*a*. *MDG (1L)*	*n*.*a*. *MBG (1L)*	*Uniform*: *U-*^*13*^*C*,^*15*^*N MBG (1 L)*	*U-*^*15*^*N*, *1*,*3-*^*13*^*C-glycerol MGG (1 L)*	*U-*^*15*^*N*, *2-*^*13*^*C-glycerol MGG (1 L)*	^*2*^*H MDG (1 L D*_*2*_*O)*	^*2*^*H*,^*15*^*N MBG (1 L D*_*2*_*O)*	^*2*^*H*,^*13*^*C*,^*15*^*N MBG (1 L D*_*2*_*O)*
2 mM MgSO_4_	2 mM MgSO_4_	2 mM MgSO_4_	2 mM MgSO_4_	2 mM MgSO_4_	2 mM MgSO_4_	2 mM MgSO_4_	2 mM MgSO_4_
0.2X trace metals mix^1^	0.2X trace metals mix^1^	0.2X trace metals mix^1^	0.2X trace metals mix^1^	0.2X trace metals mix^1^	0.2X trace metals mix^1^	0.2X trace metals mix^1^	0.2X trace metals mix^1^
0.5% w/v glucose	0.5% w/v glucose	0.5% w/v ^13^C D-glucose	0.5% w/v 1,3-^13^C glycerol	0.5% w/v 2-^13^C glycerol	0.5% w/v ^2^H-glucose	0.5% w/v ^2^H-glucose	0.5% w/v ^2^H,^13^C glucose
0.25% aspartate	10 mL 10X n.a. BioExpress	10 mL 10X U-^13^C,^15^N BioExpress	0.25% w/v ^12^C sodium carbonate	0.25% w/v ^13^C sodium carbonate	0.25% aspartate	10 mL 10X U-^2^H,^15^N BioExpress	10 mL 10X U-^2^H,^13^C,^15^N BioExpress
25 mM Na_2_HPO_4_ anhydrous	25 mM Na_2_HPO_4_ anhydrous	25 mM Na_2_HPO_4_ anhydrous	25 mM Na_2_HPO_4_ anhydrous	25 mM Na_2_HPO_4_ anhydrous	25 mM Na_2_HPO_4_ anhydrous	25 mM Na_2_HPO_4_ anhydrous	25 mM Na_2_HPO_4_ anhydrous
25 mM KH_2_PO_4_	25 mM KH_2_PO_4_	25 mM KH_2_PO_4_	25 mM KH_2_PO_4_	25 mM KH_2_PO_4_	25 mM KH_2_PO_4_	25 mM KH_2_PO_4_	25 mM KH_2_PO_4_
5 mM Na_2_SO_4_	5 mM Na_2_SO_4_	5 mM Na_2_SO_4_	5 mM Na_2_SO_4_	5 mM Na_2_SO_4_	5 mM Na_2_SO_4_	5 mM Na_2_SO_4_	5 mM Na_2_SO_4_
50 mM NH_4_Cl	50 mM NH_4_Cl	50 mM ^15^NH_4_Cl	50 mM ^15^NH_4_Cl	50 mM ^15^NH_4_Cl	50 mM NH_4_Cl	50 mM ^15^NH_4_Cl	50 mM ^15^NH_4_Cl
100 μg/mL kanamycin	100 μg/mL kanamycin	100 μg/mL kanamycin	100 μg/mL kanamycin	100 μg/mL kanamycin	100 μg/mL kanamycin	100 μg/mL kanamycin	100 μg/mL kanamycin

^1^ Trace Metals Mix from Studier et al. [20]

### Expression of triply-labeled (^2^H,^13^C,^15^N) recombinant sTF

Triply-labeled (^2^H,^13^C,^15^N) sTF samples were prepared following a modified expression protocol. The cells were conditioned to grow and express in D_2_O by slowly ramping up the fraction of D_2_O in the medium. LB (5 mL) containing 100 ug/mL kanamycin was inoculated with the sTF glycerol plasmid stock and shaken for 7 hours at 37°C at 300 rpm. The LB growth (1 mL) was inoculated into 25 mL of ~80% deuterated MDG and shaken for 12 hours at 37°C at 300 rpm. Then, 1 mL of the ~80% deuterated MDG was inoculated into 25 mL of 90% deuterated MDG and shaken for ~12 hours at 37°C at 300 rpm. The ~90% deuterated MDG culture (10 mL) was added to 500 mL of 100% deuterated MDG and divided between two 2-L baffled flasks and grown for ~15 hours at 37°C at 300 rpm until an A_600_ of ~4–5 was reached.

The cell culture was centrifuged for 15 minutes at 20°C at 6500 x g prior to being resuspended in 1 L of ~100% deuterated MBG. The resuspended culture was then divided into four 2-L baffled flasks and grown for ~1 hour at 25°C at 300 rpm until an A_600_ of ~3 was reached. Expression of sTF was induced with IPTG at a final concentration of 100 μM for 24 hours at 25°C. Cells were harvested by centrifugation for 15 minutes at 20°C at 7500 x g.

Other deuterated preparations were grown in this step-wise approach (from 0% deuteration to ~80% deuteration to ~90% deuteration to ~100% deuteration precultures into ~100% deuteration for expression media) in order to condition the cells for growth in deuterium prior to the introduction of ^13^C and ^15^N and induction of expression.

### Expression of glycerol labeled recombinant sTF using 1,3-^13^C-glycerol or 2-^13^C-glycerol

Glycerol labeled samples were prepared using a protocol highly similar to that of the uniform ^13^C,^15^N expression. Notably, in the final 250 mL MDG culture, the unlabeled glucose was replaced with natural abundance glycerol so that the cells could condition to using glycerol as the carbon source. In the expression medium, cells were grown using the modified Studier media (MGG) instead of MBG. This replaces the glucose with isotopically labeled glycerol and also excludes the BioExpress, which can only be used in uniformly labeled expressions. MGG contains 0.5% 1,3-^13^C glycerol and 0.25% ^12^C sodium carbonate or 0.5% 2-^13^C glycerol and 0.25% ^13^C sodium carbonate [18].

### Purification of isotopically labeled recombinant sTF

Frozen cell pellets from 250 mL culture were rapidly thawed at 37°C and resuspended in 20 mL of 20% sucrose in 50 mM sodium phosphate (pH 7.5) buffer, 50 mM NaCl. The cells were shaken at 4°C at 200 rpm for 10 minutes. Cells were centrifuged for 35 minutes at 4°C at 7500 x g and the supernatant was collected. The cell pellets were then rapidly resuspended in 20 mL MilliQ ultrapure water to release sTF from the cells via osmotic shock. The cells were shaken for 10 minutes at 4°C at 200 rpm. MgCl_2_ (final concentration = 1 mM) and Turbonuclease (Accelagen, San Diego, CA, USA; final concentration = 25 units/mL) were added and incubated for 1 hour at 25°C. The cells were centrifuged for 1 hour at 4°C at 8500 x g. The supernatant was combined with the supernatant from the resuspension in 20% sucrose. Q-Sepharose® Fast Flow ion exchange media (Sigma-Aldrich, St. Louis, MO, USA) were equilibrated with five changes of 50 mM sodium phosphate buffer (pH 7.5) and 50 mM NaCl (250 mL) and collected by vacuum filtration. Equilibrated Q-Sepharose beads were added to the supernatant at 1 g beads per 10 mL of supernatant and rotated for 40 minutes at 4°C. The supernatant was collected by vacuum filtration and 0.02% NaN_3_ was added.

Ni^2+^ affinity chromatography was then used to further purify the supernatant. Two 5 mL HisTrap™ Fast Flow columns (GE Healthcare, Piscataway, NJ, USA) were equilibrated with five column volumes (50 mL) of binding buffer (25 mM sodium phosphate (pH 8) buffer, 300 mM NaCl, 20 mM imidazole (pH 8)). NaCl (final concentration = 300 mM) and imidazole, pH 8 (final concentration = 20 mM) were added to the supernatant, which was subsequently filtered using a 0.45 μm membrane filter. The supernatant was loaded on the affinity columns and the columns were washed with 5–10 changes of binding buffer to remove any weakly bound protein. sTF was eluted as a single sharp peak (monitored by A_280_) with 25 mM sodium phosphate (pH 8) buffer, 300 mM NaCl, 500 mM imidazole (pH 8). Pure sTF was concentrated using Centriprep™ centrifugal filter concentrators (EMD Millipore, Billerica, MA, USA) with a molecular weight cutoff of 10 kDa. The concentrated sTF was dialyzed against three changes of 50 mM sodium phosphate (pH 7.5) buffer, 50 mM NaCl, 0.01% NaN_3_ (1.33 L) to eliminate excess imidazole.

Yields of uniform ^13^C,^15^N sTF were 60–100 mg protein per liter of media. Yields of deuterated 1,3-^13^C glycerol and 2-^13^C glycerol sTF were >80 mg protein per liter media. Thus, deuteration and/or glycerol growth did not seem to adversely impact the purified protein yield. SDS-PAGE gel electrophoresis was performed using 12% acrylamide gels run at a constant voltage of 120 V for 90 minutes with Tris-glycine running buffer.

### FVIIa amidolytic assay

The cofactor activity of sTF was measured throughout the purification using the fVIIa amidolytic assay as previously described [5]. Briefly, 25 mM HEPES (pH 7.4), 100 mM NaCl, 5 mM CaCl_2_, 0.1% bovine serum albumin, 5 nM fVIIa (Sekisui Diagnostics, Lexington, MA, USA) and varying concentrations of sTF were added to each well of a 96-well plate. The reaction began upon addition of 1 mM Chromozym t-PA substrate (Roche Diagnostics, Indianapolis, IN, USA) to each well and the rate of substrate hydrolysis was quantified by measuring the change in A_405_ for 20 minutes at 25°C.

### Solution NMR spectroscopy

Purified sTF samples were dialyzed using Slide-A-Lyzer^TM^ dialysis cassettes (Life Technologies, Grand Island, NY, USA) with a molecular weight cutoff of 10 kDa against three changes of 50 mM sodium phosphate (pH 6.5), 50 mM NaCl (1.33 L). Following dialysis, the buffer was adjusted to contain 10% D_2_O and 0.01% NaN_3_ and sTF was concentrated to ~550 μL using Centriprep™ centrifugal filter concentrators. Solution NMR experiments were performed on 100 μM uniform ^13^C,^15^N sTF in 50 mM sodium phosphate (pH 6.5), 50 mM NaCl, 5% DSS, 10% D_2_O, 0.1% NaN_3_. Spectra were acquired at a variable temperature set point of 35°C on a Varian/Agilent VNMRS 17.6 T (750 MHz ^1^H frequency) spectrometer. Spectra were processed with NMRPipe [21] and analyzed in Sparky [22].

### Magic-angle spinning (MAS) solid-state NMR spectroscopy

Two methods were used to precipitate purified sTF for solid-state NMR. The first used polyethylene glycol (PEG), in which sTF samples were precipitated after dialysis with 5–20% PEG 3350 in 25 mM sodium phosphate (pH 7.5) buffer for ~12 hours at 20°C. The second used ammonium sulfate, in which sTF was precipitated with ammonium sulfate (final concentration = ~1.8 M) in 100 mM HEPES (pH 7.5) buffer, 200 mM NaCl, 5 mM Cu-EDTA. The precipitated material was incubated for ~12 hours at 4°C and then ultracentrifuged for 2 minutes at 25°C at 10,000 x g.

Samples were packed into 3.2 mm standard and limited speed NMR rotors (Agilent Technologies, Santa Clara, CA, USA). Spectra were acquired on a Varian/Agilent VNMRS 11.7 T (500 MHz ^1^H frequency) spectrometer with a ^1^H-^13^C-^15^N 3.2 mm Balun T3 probe and a Varian/Agilent VNMRS 17.6 T (750 MHz ^1^H frequency) spectrometer with ^1^H-^13^C-^15^N 3.2 mm BioMAS probe [23]. 2D ^13^C-^13^C correlation spectra were acquired at variable temperature set points of either 0°C or -5°C and used tangent ramped cross polarization [24] with 73 kHz of ^1^H SPINAL-64 decoupling [25] and DARR mixing [26]. 2D ^15^N-^13^C correlation spectra were acquired at a variable temperature set point of -5°C and used tangent ramped cross polarization [24] with 72 kHz of ^1^H SPINAL-64 decoupling [25]. Spectra were processed with NMRPipe [21] and analyzed in Sparky [22].

## Results and Discussion

### Isotopically labeled sTF expression & purification

**Fig 1:**
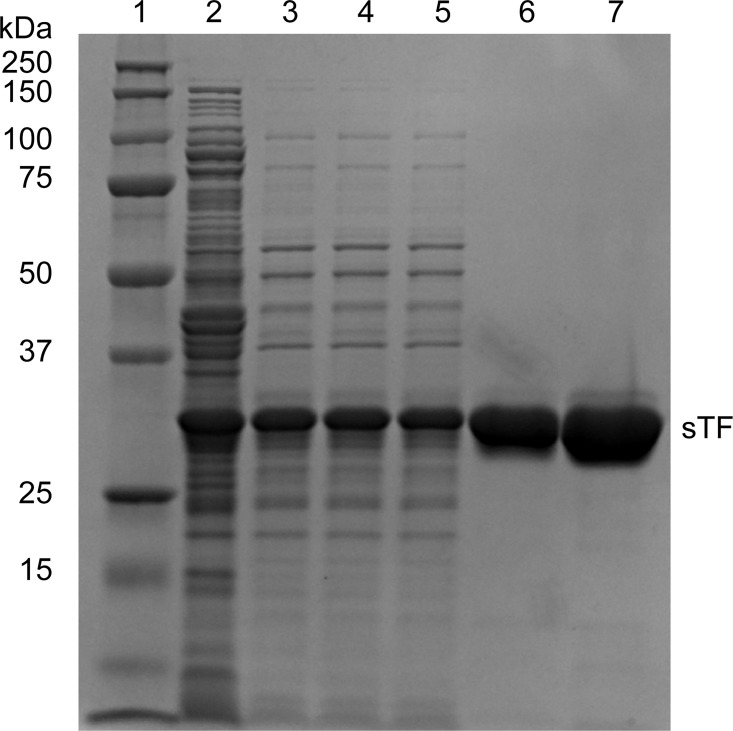
SDS-PAGE analysis of sTF purification. SDS-PAGE 12% acrylamide gel of sTF samples stained with Coomassie Brilliant Blue R-250 showing: Lane 1: Precision Plus Protein^TM^ Dual Color marker (Bio-Rad, Hercules, CA, USA); Lane 2: water and sucrose combined supernatant; Lane 3: Q-Sepharose® Fast Flow supernatant; Lane 4: Post 0.45 μm filter; Lane 5: pre-load on Ni^2+^ affinity column; Lane 6: 500 mM imidazole elution; Lane 7: concentrated sTF.

A previous sTF expression and purification protocol required protein refolding from *E*. *coli* inclusion bodies [9]. In our protocol, we utilized MDG media in order to facilitate *E*. *coli* growth to high optical density and produce healthy cultures that are stable for day-long time periods, leading to a robust sTF expression method without the need for carefully monitoring cell growth in the mid-log phase. Growing cells in MDG to saturation (A_600_ of ~5–7) produced high cell density, which yielded high expression upon IPTG induction when the bacteria were resuspended in fresh isotopically labeled media. This technique boosted protein yields while minimizing the use of expensive isotopically labeled reagents. Expression of sTF in the periplasmic space allows for native folding and disulfide bond formation, thereby eliminating the need for protein refolding [27]. Additionally, this method enabled improvement in yields of up to 100 mg per liter of uniform ^13^C,^15^N, pure sTF. This is a factor of 100 increase in yield from our previously published natural abundance sTF expression and purification method [27]. This is a 50% increase in yield of ^15^N labeled sTF from the refolding protocol in Stone et al. [9]. Yields of glycerol labeled sTF samples have not been reported previously in the literature. The relative purity at each phase of the purification is illustrated in the SDS-PAGE gel of sTF in Fig 1, indicating a very high level of purity in the concentrated sTF sample (Lane 7). Purified sTF retained full cofactor activity towards fVIIa, as measured by the fVIIa amidolytic activity assay [5], with a percent activity of 101.0 ± 3.9% relative to a standard sTF sample prepared as described previously [27,28]. The described sample preparation approach of sTF does not allow for glycosylation of the carbohydrate chains of TF. It has been shown that the carbohydrate chains are dispensable for TF function [29], although there remains some controversy in the literature as to the contribution of the carbohydrate chains to TF function [30, 31].

### Analysis of solution and solid-state NMR spectra

**Fig 2:**
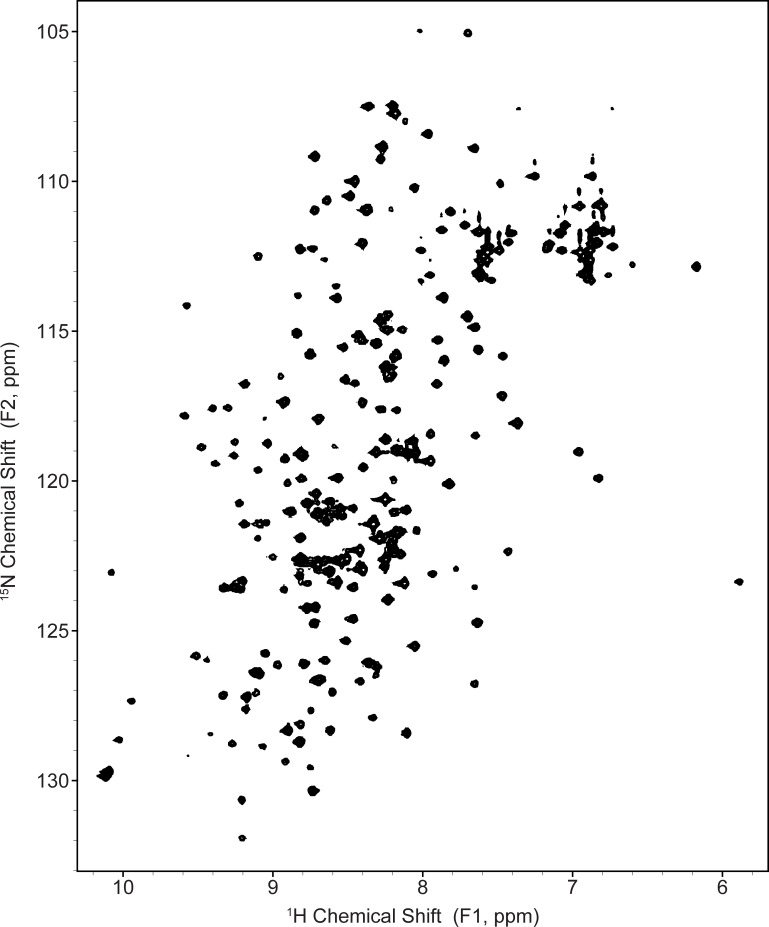
^1^H-^15^N 2D HSQC correlation spectrum. Solution NMR fingerprint spectrum of 100 μM uniform ^13^C,^15^N sTF in 50 mM sodium phosphate (pH 6.5), 50 mM NaCl, 5% DSS, 10% D_2_O, 0.1% NaN_3_ acquired at a variable temperature set point of 35°C on a Varian/Agilent VNMRS 17.6 T (750 MHz ^1^H frequency) spectrometer.

The ^1^H-^15^N 2D HSQC correlation spectrum of 100 μM sTF is shown in Fig 2. Previously, our group assigned the majority of the resonances using ~1 mM sTF samples [11]; however, sample lifetime is enhanced by utilizing 100 μM sTF concentrations, and the relative signal intensities throughout the spectrum are more consistent than the 1 mM sTF sample, indicating the presence of a pure, monodisperse sample. The fingerprint spectrum shown in Fig 2 demonstrates a common fold with prior solution NMR studies [9, 11].

Solid-state NMR samples of sTF were prepared by precipitation with either PEG or ammonium sulfate. PEG offers the advantage of lower ionic strength in the NMR sample, whereas ammonium sulfate may compromise performance for certain types of NMR probes [23]. However, the PEG-precipitated samples yielded substantially lower quantities of protein in the pellet (packed in the NMR rotor), as determined by quantitative ^13^C NMR, and showed high variability in the yield. Specifically, the PEG-precipitated samples varied from 5% to 20% sTF by mass in the pellet. In contrast, the ammonium sulfate-precipitated samples yielded ~70% sTF by mass in the pellet, and several preparations of this form showed a consistent yield.

**Fig 3:**
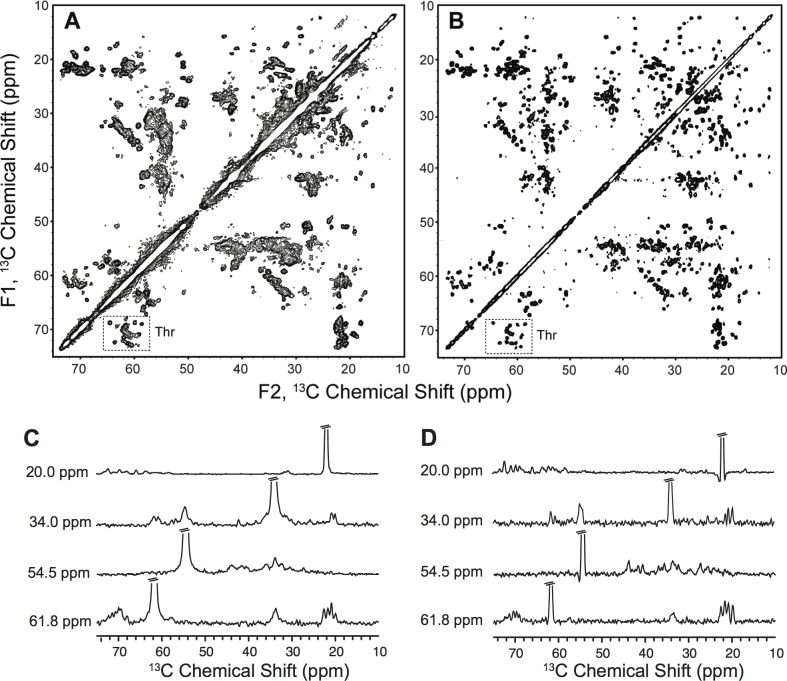
Solid-state ^13^C-^13^C 2D correlation spectra of uniform ^13^C,^15^N sTF. (A) ^13^C-^13^C 2D correlation spectrum with 25 ms of DARR mixing of ~15 mg uniform ^13^C,^15^N sTF precipitated with 15% PEG 3350 acquired at 750 MHz (^1^H frequency) for 12 hours with a MAS rate of 12.5 kHz at a variable temperature set point of 0°C. (B) ^13^C-^13^C 2D correlation spectrum with 50 ms of DARR mixing of ~31 mg uniform ^13^C,^15^N sTF precipitated with 1.8 M ammonium sulfate and 5 mM Cu-EDTA acquired at 750 MHz (^1^H frequency) for 24 hours with a MAS rate of 12.5 kHz at a variable temperature set point of -5°C. Both spectra were processed with 20 Hz of Lorentzian-to-Gaussian line broadening in each dimension, sine bell apodization, and zero filled 8192 points in the direct (F1) dimension and 4096 in the indirect (F2) dimension with contours drawn at 6 times the noise floor. (C) ^13^C 1D slices extracted from the ^13^C-^13^C 2D spectrum of PEG-precipitated sTF shown in A. The corresponding ^13^C chemical shifts in the indirect dimension are indicated. (D) ^13^C 1D slices extracted from the ^13^C-^13^C 2D spectrum of ammonium sulfate-precipitated sTF shown in B. The corresponding ^13^C chemical shifts in the indirect dimension are indicated.

**Fig 4:**
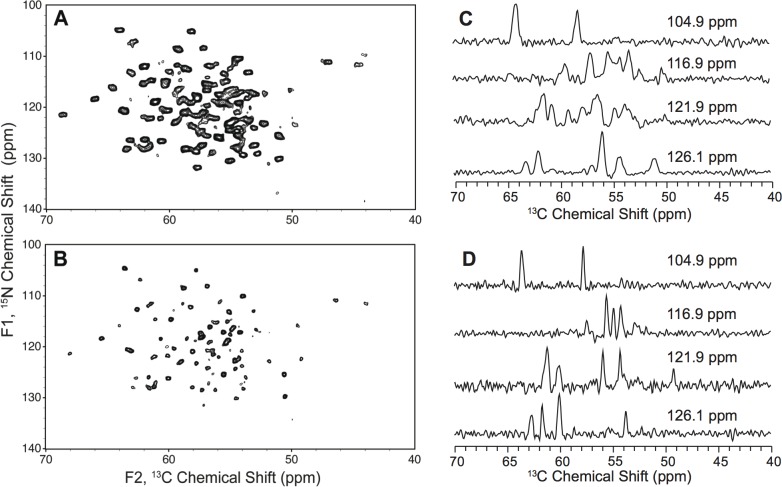
Solid-state ^15^N-^13^CA correlation spectra of uniform and glycerol labeled sTF. (A) ^15^N-^13^CA correlation spectrum of ~30 mg of uniform ^13^C,^15^N ammonium sulfate-precipitated sTF with 5 mM Cu-EDTA. The spectrum was acquired for 3 hours and 20 minutes with an acquisition time of 25 ms. (B) ^15^N-^13^CA correlation spectrum of ~25 mg of 2-^13^C glycerol, ^2^H, ^15^N ammonium sulfate-precipitated sTF with 5 mM Cu-EDTA. The spectrum was acquired for 3 hours with an acquisition time of 30 ms. Both spectra were collected at 750 MHz (^1^H frequency) at a variable temperature set point of -5°C with a MAS rate of 12.5 kHz. Both spectra were processed with 10 Hz of Lorentzian-to-Gaussian line broadening in each dimension, sine bell apodization, and zero filled 8192 points in the direct (F1) dimension and 2048 in the indirect (F2) dimension with contours drawn at 6 times the noise floor. (C) ^13^C 1D slices extracted from the ^15^N-^13^CA 2D spectrum of uniform ^13^C-^15^N ammonium sulfate-precipitated sTF shown in A. The corresponding ^15^N chemical shifts in the indirect dimension are indicated. (D) ^13^C 1D slices extracted from the ^15^N-^13^CA 2D spectrum of 2-^13^C glycerol, ^2^H, ^15^N ammonium sulfate-precipitated sTF shown in B. The corresponding ^15^N chemical shifts in the indirect dimension are indicated.

Utilizing low electric field resonators [23] allowed for the acquisition of high-resolution, multidimensional solid-state NMR data sets using the ammonium sulfate-precipitated sTF samples. The ^13^C-^13^C 2D correlation spectra with DARR mixing are shown in Fig 3. The ammonium sulfate-precipitated sample exhibits substantially better resolution than the PEG-precipitated sample, which we attribute to the greater regularity of crystal formation under these conditions. Additionally, the ammonium sulfate-precipitated sample exhibits inhomogeneous linewidths of less than 0.1 ppm, and the signal-to-noise ratios for the one-bond correlations are >30:1 in 24 hours. By comparison, the PEG-precipitated sample is less well-resolved, particularly in the Thr CA-CB region highlighted in Fig 3, which is diagnostic of sample homogeneity. Because scalar couplings contribute to the majority of the linewidth in the case of the ammonium sulfate-precipitated sample, additional enhancements in resolution are achievable by using glycerol labeling, as shown in Fig 4. In particular, 2-^13^C glycerol labeling reduced the ^13^C linewidths anywhere from 14 to 116 Hz, with most chemical shifts exhibiting ^13^C linewidth reductions of approximately 50 Hz; however, the sensitivity of the 2-^13^C glycerol ^2^H,^15^N sample is lower than that of the uniform ^13^C,^15^N sample due to a reduction in cross polarization efficiency from deuteration as well as a lower total amount of sample in the NMR rotor. Chemical shifts between the uniform ^13^C,^15^N and 2-^13^C glycerol ^2^H,^15^N sTF samples also differ slightly due to deuterium isotope effects.

Initial analyses of the ammonium sulfate-precipitated spectra reveal that chemical shift information for residues missing electron density in the highest resolution sTF crystal structure [6] are observed. For example, we observe 18 out of 18 Serine resonances in the PEG and ammonium sulfate-precipitated samples; the 2HFT structure is missing electron density for a Serine-rich functionally relevant loop. Further, 27 out of 27 Threonine resonances and 9 out of 10 Alanine resonances are observed, due to the flexibility of one Alanine residue in the N-terminal region of the protein. There are also several Glycine residues located in flexible loop regions of sTF and we observe 9 of 14 Glycine resonances. The PEG and ammonium sulfate- precipitated samples have highly overlapping chemical shifts, indicating a common fold, which also is consistent with the solution NMR data [11].

## Conclusions

NMR provides unique opportunities to investigate atomistic details of blood coagulation, including structure, interactions, and dynamics. Towards this end, we have developed greatly improved methods for expression and purification of isotopically labeled, active sTF that enable highly resolved spectra to be collected using both solution and solid-state NMR. We have also greatly increased the sensitivity of solid-state NMR measurements of sTF, by increasing the fraction of protein in the pellet using ammonium sulfate precipitation. This procedure yields a factor of at least 3 to 5 increase in sensitivity relative to the PEG precipitation, yet both forms give highly similar chemical shifts, indicating a common fold. Further, selective ^13^C-labeling of sTF greatly enhances the sensitivity as well as resolution for multidimensional solid-state NMR studies. Chemical shift data can now be compared between the soluble and crystalline forms of TF, and perturbations will shed light on the structure of sTF and ultimately the structure and mechanism of the TF/fVIIa complex.

## Supporting Information

S1 Fig Full SDS-PAGE gel of sTF purification. SDS-PAGE 12% acrylamide gel of sTF samples stained with Coomassie Brilliant Blue R-250 showing Lane 1: Precision Plus Protein^TM^ Dual Color marker (Bio-Rad, Hercules, CA, USA); Lane 2: water and sucrose combined supernatant; Lane 3: Q-Sepharose® Fast Flow supernatant; Lane 4: Post 0.45 μm filter; Lane 5: pre-load on Ni^2+^ affinity column; Lane 6: 500 mM imidazole elution; Lane 7: concentrated sTF; Lanes 8–10: empty; Lane 11: 500 mM imidazole elution; Lane 12: pre-load on Ni^2+^ affinity column; Lane 13: Post 0.45 μm filter; Lane 14: Q-Sepharose® Fast Flow supernatant; Lane 15: Precision Plus Protein^TM^ Dual Color marker (Bio-Rad, Hercules, CA, USA).
(TIF)

## References

[1] Morrissey JM. Tissue factor: a key molecule in hemostatic and nonhemostatic systems. Int J Hem. 2004;79: 103–108. 10.1532/ijh97.03167 15005335

[2] Drake TA, Morrissey JH, Edgington TS. Selective cellular expression of tissue fctor in human tissues- implications for disorders of hemostasis and thrombosis. Am J Pathol. 1989;134: 1087–1097. 2719077 PMC1879887

[3] Ruf W, Rehemtulla A, Morrissey JH, Edginton TS. Phospholipid-independent and dependent interactions required for tissue factor receptor and cofactor function. J Biol Chem. 1991;266: 2158–2166. 1989976

[4] Carlsson K, Perrson E, Lingren M, Carlsson U, Svensson M. Effects on the conformation of FVIIa by sTF and Ca^2+^ binding: studies of fluorescence resonance energy transfer and quenching. Biochem Bioph Res Co. 2011;413: 545–549. 10.1016/j.bbrc.2011.08.135 21924243

[5] Neuenschwander PF, Branam DE, Morrissey JH. Importance of substrate composition, pH and other variables on tissue factor enhancement of factor VIIa activity. Thromb Haemostasis. 1993;70(6): 970–977. 8165620

[6] Muller YA, Ultsch MH, Kelley RF, de Vos AM. Structure of the extracellular domain of human tissue factor: location of the factor VIIa binding site. Biochemistry-US. 1994;33: 10864–10870. 10.1021/bi00202a003 8086403

[7] Harlos K, Martin DMA, O'Brien DP, Jones EY, Stuart DI, Polikarpov I, et al Crystal structure of the extracellular region of human tissue factor. Nature. 1994;370: 662–666. 8065454 10.1038/370662a0

[8] Huang M, Syed R, Stura EA, Stone MJ, Stefanko RS, Ruf W, et al The mechanism of an inhibitory antibody on TF-initiated blood coagulation revealed by the crystal structures of human tissue factor, Fab 5G9 and TF.G9 complex. J Mol Biol. 1998;275: 873–894. 9480775 10.1006/jmbi.1997.1512

[9] Stone MJ, Ruf W, Miles DJ, Edgington TS, Wright PE. Recombinant soluble human tissue factor secreted by Saccharomyces cerevisiae and refolded from Escherichia coli inclusion bodies: glycosylation of mutants, activity and physical characterization. Biochem J. 1995;310: 605–614. 7654202 10.1042/bj3100605 PMC1135939

[10] Monroe DM, Key NS. The tissue factor-factor VIIa complex: procoagulant activity, regulation, and multitasking. J Thromb Haemost. 2007;5: 1097–1105. 17567444 10.1111/j.1538-7836.2007.02435.x

[11] Boettcher JM, Clay MC, LaHood BJ, Morrissey JH, Rienstra CM. Backbone ^1^H, ^13^C and ^15^N resonance assignments of the extracellular domain of tissue factor. Biomol NMR Assigm. 2010;4: 183–185. 10.1007/s12104-010-9233-x PMC2947601 20526825

[12] Sperling LJ, Berthold DA, Sasser TL, Jeisy-Scott V, Rienstra CM. Assignment strategies for large proteins by magic-angle spinning NMR: the 21-kDa disulfide-bond-forming enzyme DsbA. J Mol Biol. 2010;399: 268–282. 10.1016/j.jmb.2010.04.012 20394752 PMC2880403

[13] Gardiennet C, Schutz AK, Hunkeler A, Kunert B, Terradot L, Bockmann A, et al A sedimented sample of a 59 kDa dodecameric helicase yields high-resolution solid-state NMR spectra. Angew Chem Int Ed. 2012;51: 7855–7858. 10.1002/anie.201200779 22740125

[14] Shi L, Lake E, Ahmed M, Brown LS, Ladizhansky V. Solid-state NMR study of proteorhodopsin in the lipid environment: secondary structure and dynamics. BBA-Biomembranes. 2009;1788: 2563–2574. 10.1016/j.bbamem.2009.09.011 19799854

[15] Gao M, Nadaud PS, Bernier MW, North JA, Hammel PC, Poirier MG, et al Histone H3 and H4 N-terminal tails in nucleosome arrays at cellular concentrations probed by magic angle spinning NMR spectroscopy. J Am Chem Soc. 2013;135: 15278–15281. 10.1021/ja407526s 24088044 PMC3856215

[16] Han Y, Ahn J, Concel J, Byeon IL, Gronenborn AM, Yang J, et al Solid-state NMR studies of HIV-1 capsid protein assemblies. J Am Chem Soc. 2010;132(6): 1976–1987. 10.1021/ja908687k 20092249 PMC2829833

[17] LeMaster DM, Kushlan DM. Dynamical mapping of *E*. *coli* thioredoxin via ^13^C NMR relaxation analysis. J Am Chem Soc. 1996;118(39): 9255–9264.

[18] Castellani F, van Rossum B, Diehl A, Schubert M, Rehbein K, Oschkinat H. Structure of a protein determined by solid-state magic-angle-spinning NMR spectroscopy. Nature. 2002;420: 98–102. 12422222 10.1038/nature01070

[19] Higman VA, Flinders J, Hiller M, Jehle S, Markovic S, Fiedler S, et al Assigning large proteins in the solid state: a MAS NMR resonance assignment strategy using selectively and extensively ^13^C-labelled proteins. J Biomol NMR. 2009;44(4): 245–260. 10.1007/s10858-009-9338-7 19609683

[20] Studier FW. Protein production by auto-induction in high density shaking cultures. Protein Expres Purif. 2005;41: 207–234. 10.1016/j.pep.2005.01.016 15915565

[21] Delaglio F, Grzesiek S, Vuister GW, Zhu G, Pfeifer J, Bax A. Nmrpipe: a multidimensional spectral processing system based on unix pipes. J Biomol NMR. 1995;6: 277–293. 8520220 10.1007/BF00197809

[22] Goddard TD, Kneller DG. (2006) Sparky 3, University of California, San Francisco.

[23] Stringer JA, Bronnimann CE, Mullen CG, Zhou DH, Stellfox SA, Li Y, et al Reduction of RF-induced sample heating with a scroll coil resonator structure for solid-state NMR probes. J Magn Reson. 2005;173: 40–48. 15705511 10.1016/j.jmr.2004.11.015

[24] Hediger S, Meier BH, Kurur ND, Bodenhausen G, Ernst RR. NMR cross-polarization by adiabatic passage through the Hartmann-Hahn Condition. Chem Phys Lett. 1994;223: 283–288.

[25] Fung BM, Khitrin AK, Ermolaev K. An improved broadband decoupling sequence for liquid crystals and solids. J Magn Reson. 2000;142: 97–101. 10617439 10.1006/jmre.1999.1896

[26] Takegoshi K, Nakamura S, Terao T. C-13-H-1 dipolar-assisted rotational resonance in magic-angle spinning NMR. Chem Phys Lett. 2001;344: 631–637.

[27] Rezaie AR, Fiore MM, Neuenschwander PF, Esmon CT, Morrissey JH. Expression and purification of a soluble tissue factor fusion protein with an epitope for an unusual calcium-dependent antibody. Protein Expres Purif. 1992;3: 453–460. 10.1016/1046-5928(92)90062-2 1283093

[28] Gajsiewicz JM, Nuzzio KM, Rienstra CM, Morrissey JH. Tissue factor residues that modulate magnesium-dependent rate enhancements of the tissue factor/factor VIIa complex. Biochemistry. 2015;54(30): 4665–4671. 10.1021/acs.biochem.5b00608 26169722 PMC4862878

[29] Paborsky LR, Tate KM, Harris RJ, Yansura DG, Band L, McCray G, et al Purification of recombinant human tissue factor. Biochemistry. 1989;28(20): 8072–8077. 2690932 10.1021/bi00446a016

[30] Butenas S, Amblo-Krudysz J, Mann KG. Posttranslational modifications of tissue factor. Front Biosci (Elite Ed). 2012;4: 381–391. 22201880 10.2741/385 PMC3247914

[31] Kothari H, Pendurthi UR, Rao LV. Tissue factor purified from different cellular sources and non-glycoslyated tissue factor show similar procoagulant activity. J Thromb Haemost. 2013;11(11): 2066–2068. 10.1111/jth.12407 24112816 PMC4174342

