# Supplementary figures and images for: High-Resolution NMR Studies of Human Tissue Factor
Source: PLoS One. 2016 Sep 22;11(9):e0163206. doi: 10.1371/journal.pone.0163206 (PMC5033421; doi:10.1371/journal.pone.0163206)

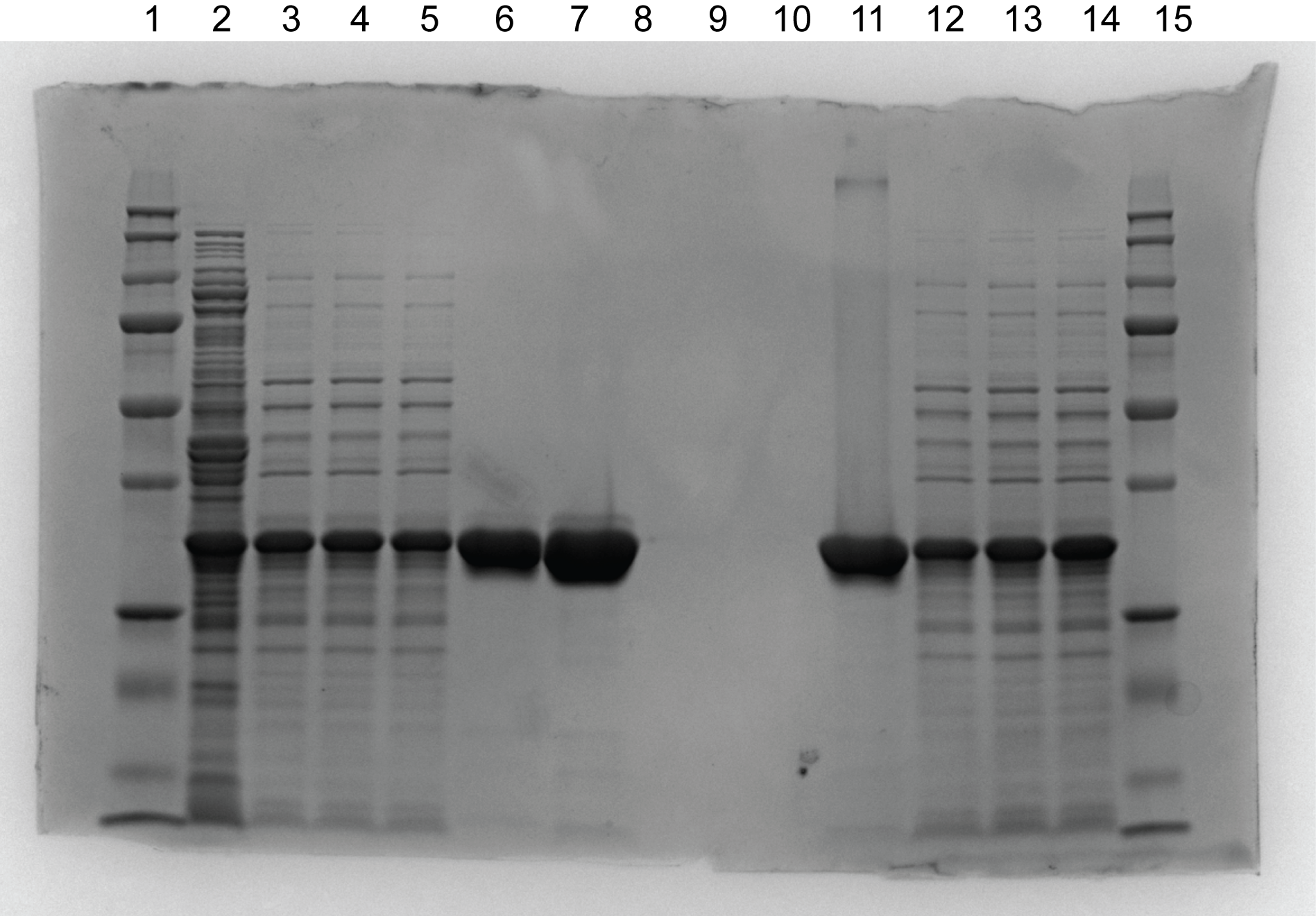

Supplement: S1 Fig — SDS-PAGE 12% acrylamide gel of sTF samples stained with Coomassie Brilliant Blue R-250 showing Lane 1: Precision Plus ProteinTM Dual Color marker (Bio-Rad, Hercules, CA, USA); Lane 2: water and sucrose combined supernatant; Lane 3: Q-Sepharose® Fast Flow supernatant; Lane 4: Post 0.45 μm filter; Lane 5: pre-load on Ni2+ affinity column; Lane 6: 500 mM imidazole elution; Lane 7: concentrated sTF; Lanes 8–10: empty; Lane 11: 500 mM imidazole elution; Lane 12: pre-load on Ni2+ affinity column; Lane 13: Post 0.45 μm filter; Lane 14: Q-Sepharose® Fast Flow supernatant; Lane 15: Precision Plus ProteinTM Dual Color marker (Bio-Rad, Hercules, CA, USA). (TIF) [file pone.0163206.s001.tif]
